# Proprioception: A New Era Set in Motion by Emerging Genetic and Bionic Strategies?

**DOI:** 10.1146/annurev-physiol-040122-081302

**Published:** 2022-11-18

**Authors:** Paul D. Marasco, Joriene C. de Nooij

**Affiliations:** 1 Laboratory for Bionic Integration, Department of Biomedical Engineering, Lerner Research Institute, Cleveland Clinic, Cleveland, Ohio, USA; 2 Charles Shor Epilepsy Center, Cleveland Clinic, Cleveland, Ohio, USA; 3 Advanced Platform Technology Center, Louis Stokes Cleveland Department of Veterans Affairs Medical Center, Cleveland, Ohio, USA; 4 Department of Neurology and the Columbia University Motor Neuron Center, Columbia University Medical Center, New York, NY, USA

**Keywords:** proprioceptive sensory neurons, muscle spindle, Golgi tendon organ, kinesthetic illusion, dorsal column nuclei, somatosensory cortex, neural machine interface, prosthetic limb

## Abstract

The generation of an internal body model and its continuous update is essential in sensorimotor control. Although known to rely on proprioceptive sensory feedback, the underlying mechanism that transforms this sensory feedback into a dynamic body percept remains poorly understood. However, advances in the development of genetic tools for proprioceptive circuit elements, including the sensory receptors, are beginning to offer new and unprecedented leverage to dissect the central pathways responsible for proprioceptive encoding. Simultaneously, new data derived through emerging bionic neural machine–interface technologies reveal clues regarding the relative importance of kinesthetic sensory feedback and insights into the functional proprioceptive substrates that underlie natural motor behaviors.

## INTRODUCTION

Proprioception is the sense of the movement and position of the body and limbs in space (1). It derives from dedicated peripheral proprioceptive receptors, the cell bodies of which reside in dorsal root ganglia, trigeminal ganglia, and the mesencephalic trigeminal nucleus. Proprioceptive neurons are pseudounipolar with a single axon that extends from the cell body and splits into two branches. One branch innervates peripheral receptive tissues to read changes in body position, while the other projects toward neural targets in the spinal cord to relay this information onto spinal or supraspinal sensory-motor circuits. Proprioceptive feedback is transmitted to spinal reflex circuits to help stabilize posture, coordinate state transitions between limb positions to ensure fluidic movements, or enable adaptations to the motor plan when unexpected factors derail an intended motor goal (2–4). At the same time, proprioceptive information from the periphery is routed to supraspinal sensory and motor centers, including cortex, where it informs a consciously accessible (yet not always attended to) internal model of the movements and positions of the body and limbs. A continuously updated internal body model contributes to motor planning and learning. This internal body model also helps form the basis of a sense of the embodied self that is separate from others and the external world.

We have gained an appreciation for the essential role that proprioception fulfils in most if not all aspects of motor control, yet its function beyond the level of simple spinal reflexes remains poorly understood and much debated (2, 3, 5). Two developments are giving a new impetus for settling these discussions. Despite an incomplete understanding of the proprioceptive system at a circuit level, the integration of proprioceptive feedback has become increasingly relevant in the design of prosthetic limbs. Although initially centered on feedback from cutaneous receptors (6, 7), advanced rehabilitation technologies are now incorporating closed-loop proprioceptive feedback into their designs (8–18). Such strategies are providing a window into the features of proprioception that are salient to functional recovery while also offering a new systems-level perspective on how proprioception may update internal body maps.

Simultaneously, the transcriptomic age has caught up with the proprioceptive field and offers unprecedented insight into the transcription factors, membrane receptors, and ion channels that shape the identities of proprioceptive neurons across spine, thalamus, and cortex (19–24). Along with advances in mouse genetic and viral resources, unique transcriptional insights into proprioceptor cell types offer new genetic strategies to test or refine new hypotheses regarding the role of proprioception in motor control and the sense of self-versus-other. In line with our own areas of expertise, this review highlights some of these new developments against the backdrop of the present knowledge and questions related to the peripheral and central neural elements that underlie the proprioceptive sense. In an effort to include more recent publications in our discussion, we focus less on the foundational studies that have propelled this field.

## RELEVANT SOURCES AND TYPES OF PROPRIOCEPTIVE FEEDBACK

**Figure 1:**
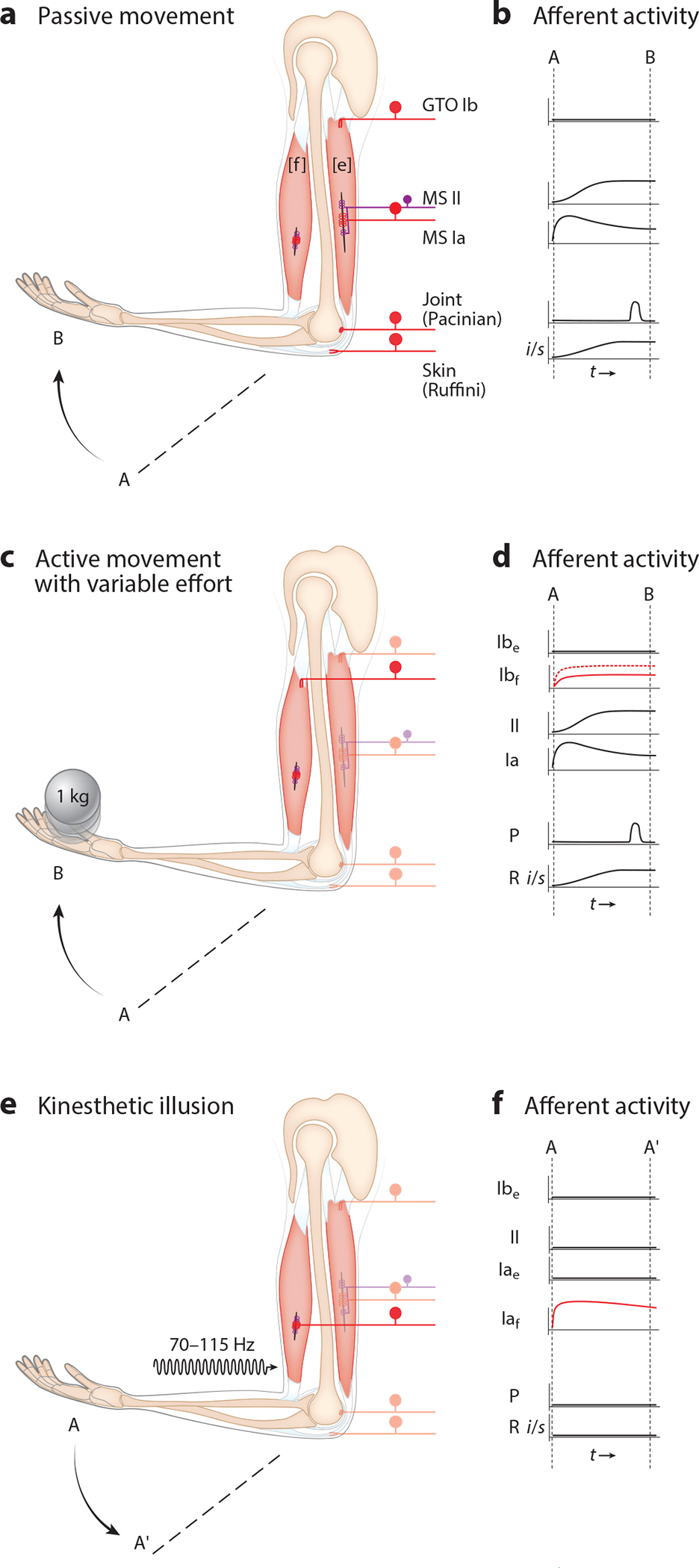
Proprioceptive sensory neurons and their movement-evoked activity patterns. (*a*, *c*, *e*) Schematic renderings of elbow flexor ([f]) and extensor ([e]) muscles with innervation by group Ia and II muscle spindle (MS) afferents and group Ib Golgi tendon organ (GTO) afferents, elbow joint Pacinian (P) afferents, and elbow skin Ruffini (R) afferents. (*b*, *d*, *f*) Fictive electrophysiological traces representing afferent activities [impulses/second (*i*/*s*)] during basic (*a*) passive, (*c*) active, and (*e*) illusionary movements. During passive elbow flexion from position A to B (*a*, *b*), muscle spindle afferents, but not GTO afferents, are activated within the stretched elbow extensor. Rapidly adapting Pacinian afferents only show temporal activity near the maximum extend of joint rotation, while slowly adapting Ruffini endings in skin gradually increase their activity with increasing skin stretch. During active elbow flexion from position A to B (*c*, *d*), increased muscle tension in the contracting flexor muscle scales with increased activity of group Ib GTO afferents. In panel *d*, the solid lines represent group Ib activity in the absence of added weights (tension); dashed lines represent group Ib activity in the presence of added weights. Activities of extensor afferents are essentially the same as in panel *b*. Stimulation of the elbow flexor muscle tendon (*e*) with vibration in the 70–115 Hz frequency bandwidth can evoke the illusion of elbow extension from position A to A. The kinesthetic illusion is thought to be mediated by the activity of group Ia muscle spindle afferents (*f*); activity of other afferents is unchanged for the arm that remains stationary.

Proprioception includes the senses of joint and body position, the kinesthetic sense of their movement through space, and the sense of muscle force (2–5). Any tissue imbued with mechanical sensors that exhibits a change in shape or tension as a consequence of passive or self-generated motor actions may serve as a source of proprioceptive information. By this definition, proprioceptors include afferents that innervate skeletal muscle and tendons, joint ligaments, connective tissues surrounding muscles, and skin (Figure 1a).

Muscle proprioceptors include muscle spindle and Golgi tendon organ (GTO) afferents (25, 26) (Figure 1a,b). Muscle spindles are encapsulated sensory end organs that consist of specialized intrafusal muscle fibers innervated by primary (group Ia) and secondary (group II) proprioceptive neurons (for a recent review on muscle spindle structural features and associated afferents, see 27). Both afferent types are responsive to stretch of the intrafusal fibers, such that voluntary or passive changes in limb position (with muscle length as the readout) result in increased or decreased firing rates (28, 29). Muscle spindle group Ia afferents, because of their relative ease of physiological access, have long been the proprioceptor of choice for experimental examination, perhaps leading to an outsized view of their relevance in sensorimotor control.

GTO afferents represent a second class of proprioceptive muscle afferents. They are large-caliber, low-threshold sensory neurons, termed group Ib, that innervate GTO mechanoreceptive organs that are located at myotendinous junctions, where extrafusal muscle fibers attach to tendons or aponeuroses (26). Group Ib afferents are extremely responsive to small contractions in extrafusal muscle fibers, which increase their firing rates (29–31) (Figure 1b). As such, they are primarily considered as the sensors of muscle force.

Proprioceptors also include various joint and skin receptors (Figure 1a). Joint receptors are activated at the extreme ends of joint rotation and appear to act as limit detectors; however, they likely also regulate joint/limb stiffness (3, 32, 33). Joint receptors include Pacinian/Paciniform corpuscles, Ruffini endings, and sometimes GTO afferents (34, 35). These afferents can be observed throughout the body but are considered joint receptors by virtue of their association with articular ligaments. Skin proprioception can also be mediated by the same group of sensory receptors, but in this case, they are embedded in the skin and deep connective tissues overlying moving joints or contracting muscles. Tactile sensory neurons, including Meissner and Merkel cell afferents, are activated by a movement or touch across the skin (36, 37). Skin sensation is critical in motor tasks such as prehension during object manipulation (38).

A critical feature of all proprioceptive afferents is the expression of the mechanoreceptive transduction channel Piezo2.Studies in mice demonstrated that Piezo2 localizes to the sensory endings of muscle proprioceptors and tactile receptors, and in both mouse and human, the loss of Piezo2 is associated with severe impairments in motor coordination (39, 40). Although Piezo2 is critical for the initial depolarization of most proprioceptive sensory endings, it may act in conjunction with other molecules (e.g., glutamate, voltage-gated sodium channels) to pattern the overall impulse activity of proprioceptor peripheral endings (41–43). Other basic physiological features of these muscle, joint, or skin proprioceptors are also beginning to be understood (25–27, 36), yet how their combined feedback synthesizes into a dynamic proprioceptive percept, which is needed for motor planning and adaptation, has been difficult to study.

## ASCENDING PROPRIOCEPTIVE PATHWAYS AND FEEDBACK MODULATION

Integrating and processing information from different proprioceptor subtypes hinge on convergence in ascending neural pathways. Yet, the relationship between proprioceptor subtypes and the extent to which information content changes depending on transmission route, or muscle or body segment of origin, have proven difficult to disentangle with available technologies. With a look toward bridging peripheral proprioceptive receptors and cortical representations of limb dynamics in future genetic experiments, we provide an overview of the main anatomical trajectories through which proprioceptive sensory feedback reaches higher-order processing centers.

**Figure 2:**
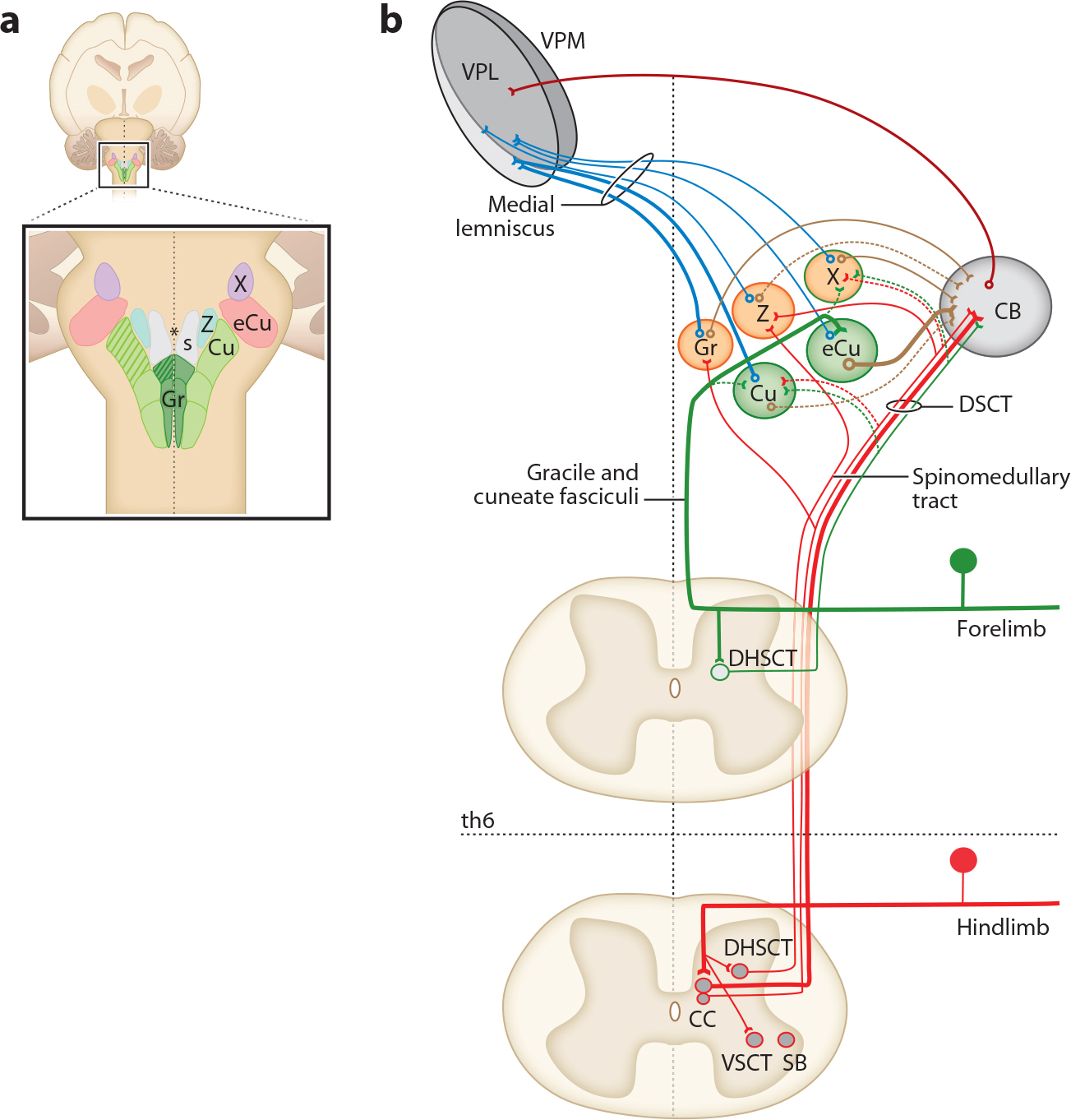
Ascending proprioceptive pathways. (*a*) The DCN complex is located in the medulla and consists of multiple nuclei that are bilaterally positioned near the obex (*). The core dorsal column nuclei are formed by the Gr and Cu nuclei; the DCN complex also includes the eCu nucleus, nucleus Z, and nucleus X (44). All DCN complex nuclei relay proprioceptive information from muscle (muscle spindles and Golgi tendon organs) and deep tissues (joints, tendons, skin), with the exception of eCu, which appears mostly restricted to muscle afferent feedback. Within Gr (hindlimb) and Cu (forelimb and neck) nuclei, proprioceptor-responsive neurons (including joint receptors) are mainly confined to the rostral domains (*hatched area*) (44, 65). (*b*) Ascending proprioceptive muscle afferent trajectories from forelimb (*green*) and hindlimb (*red*) levels. Muscle afferent feedback from the neck, forelimb, and upper thoracic segments reaches the DCN complex via the direct (Gr and Cu fasciculi) and indirect pathways (through collaterals of the DSCT and spinomedullary tract). Muscle afferent feedback from below th6 segments and hindlimb reaches the DCN complex only through the indirect DCN pathway. Note that feedback from hindlimb cutaneous proprioceptors to the gracile nucleus is relayed through the direct pathway (Gr fasciculus; not shown). The extent to which collaterals from DSCT projecting axons innervate multiple DCN complex nuclei remains poorly characterized (but see 48). Ascending DCN complex projections to the VPL thalamic nucleus and CB are represented by blue and brown lines, respectively; CB to VPL projections are represented by a dark red line. Line format (solid versus dashed, thin versus thick) indicates relative prominence of connections as globally observed across rodents, cats, and nonhuman primates (44). Abbreviations: CB, cerebellum; CC, Clarke’s column; Cu, cuneate; DCN, dorsal column nuclear; DHSCT, dorsal horn spinal cerebellar tract neurons; DSCT, dorsal spinocerebellar tract; eCu, external cuneate; Gr, gracile; S, solitary nucleus; SB, spinal border cell nucleus; th6, mid-thoracic segment 6; VPL, ventroposterior lateral nucleus; VPM, ventroposterior medial nucleus; VSCT, ventral spinal cerebellar tract neurons; X, nucleus X; Z, nucleus Z.

The neural architecture for ascending proprioceptive pathways is broadly consistent across rodents, cats, nonhuman primates, and humans (44). Proprioceptive information primarily reaches the cortex through the cerebello-thalamo-cortical pathway and the dorsal column–medial lemniscus cortical pathway (44–46). The first relay stations in both pathways are the ascending second-order spinal projection neurons that transmit sensation to the cerebellum and/or brain stem dorsal column nuclear (DCN) complex (Figure 2a).These spinal ascending neurons primarily project through the dorsal columns (cuneate and gracilis fasciculi), the dorsal spinocerebellar tract (DSCT), the spinomedullothalamic tract (traveling through the dorsolateral funiculus), and the ventral spinocerebellar tract (traveling through the ventral funiculus) (Figure 2b). Spinal projection neurons that transmit proprioceptive information to supraspinal nuclei in the medulla also include the spino-to-lateral reticulus neurons and the spino-olivary tract neurons (47, 48). Although the lateral reticulus and inferior olive also have important sensory-motor control functions (49, 50), here, we focus on the cerebellar and DCN complex pathways.

### Cerebello-Thalamo-Cortical Pathway

Anatomical and electrophysiological studies of proprioceptive spinocerebellar projection neurons indicate the existence of multiple subtypes distributed along the rostral caudal extent of the spinal cord (24, 51, 52). Some are grouped into discrete clusters (e.g., central cervical nucleus, cervical LVII neurons, Clarke’s column, spinal border cell nucleus), while others appear more randomly distributed. Many proprioceptive spinocerebellar projection neurons link directly to the cerebellum through dedicated tracts that terminate as mossy fibers on granule cells (Figure 2b) (52, 53). Proprioceptive information also reaches the cerebellum through second-order projection neurons within the DCN complex through the cuneocerebellar pathway (44) (Figure 2b).

Owing to the heterogeneity of spinocerebellar tract (SCT) neurons, how qualitatively distinct proprioceptive information flows across the various spinocerebellar projection neurons remains poorly understood. Nevertheless, electrophysiological analyses indicate that SCT neurons can relay information from various receptor types, including cutaneous mechanoreceptors (51, 54–57). Consistent with their wide-ranging inputs, SCT neurons (e.g., the central cervical nucleus and Clarke’s column) are thought to encode whole limb kinematics rather than features of individual muscles (55, 56, 58), although it is unclear whether this is true for all SCT neurons. Less is known about the more randomly distributed SCT neurons, but some may constitute cerebellar projection neurons that collateralize to the DCN complex (unlike Clarke’s column neurons) (47, 48). Recent transcriptomic studies have begun to provide molecular signatures for many of the different SCT neurons (24, 48). It is likely that this work will yield new genetic opportunities to examine SCT neuron input/output selectivity.

Within cerebellum, proprioceptive sensory feedback is thought to be assessed against a dynamic forward body model constructed on the basis of motor efference copies (59). Sensory feedback that deviates from the predicted forward model is extracted as an error/suggestions for improvement signal and—among other targets—relayed back to cortex through the deep cerebellar nuclei and thalamus (45, 60, 61). Considering this cerebellar role, it may make sense that Purkinje neurons could only need sensory information about global limb kinetics such as that provided by Clarke’s column inputs. However, some Purkinje neurons may multiplex and simultaneously encode muscle- or joint-specific features (62).

### Dorsal Column-Medial Lemniscus-Cortical Pathway

Proprioceptive information is relayed through most nuclei of the DCN complex, including gracile, cuneate, external cuneate, nucleus Z, and nucleus X (44). Within the gracile and cuneate nuclei, muscle proprioception is confined to the rostral and ventral domains (63–65) (Figure 2a). Although rostral gracile and cuneate neurons also show responses to low-threshold cutaneous neurons, cutaneous feedback primarily maps to the mid and caudal domains. Afferents with larger receptor fields (e.g., from Pacinian corpuscles) are mostly represented within the caudal domains of these nuclei (44, 64–66). In contrast to all other DCN complex nuclei, external cuneate is thought to receive exclusively noncutaneous muscle-sensory information, although previous studies reported some non-Pacinian rapidly adapting responses (63, 64).

The distribution of proprioceptive feedback across the DCN complex follows a roughly topographic organization, with rostroventral cuneate and external cuneate biased to transmitting feedback from neck and forelimb levels, and rostroventral gracile and nucleus Z mostly relaying information from lower limb and axial muscles (44, 63, 65, 67–69) (Figure 2b). Nucleus X receives proprioceptive (and cutaneous) inputs from both the fore- and hindlimb. Afferent information reaches the DCN complex through either the direct dorsal column pathway or collaterals from the spinocerebellar and/or spinomedullary tracts in the dorsal lateral funiculus (67, 70) (Figure 2b). Recordings from DCN complex neurons (including cuneate, external cuneate, nucleus X, and nucleus Z) demonstrate that the majority of neurons exhibit selective responses for individual muscles, with a few showing convergent input from multiple muscles acting at the same joint (64, 67, 71–74). The predominantly selective muscle responses in DCN complex neurons suggest that DSCT neurons collateralizing to gracile, cuneate, nucleus Z, or nucleus X transmit more selective information than Clarke’s column DSCT neurons. The functional relevance of the complex organization of the input trajectories to the DCN complex is not yet understood but is beginning to be explored with genetic tools (47, 48).

Several studies have mapped receptor-specific DCN inputs that distinguished between group Ia muscle spindles, group II muscle spindles, Ib GTO afferents, and Pacinian afferents (64, 71). Responses from all muscle afferents were observed in cuneate, external cuneate, and nucleus Z, with many neurons responding to either group Ia or group Ib stimulation but not to both (71, 73). These data suggest that for many DCN complex nuclei, proprioceptive inputs are both muscle and submodality specific. Recent observations suggest that, with respect to tactile inputs, cuneate also performs significant subcortical preprocessing such that its output can resemble a multimodality representation of touch similar to observations in cortex (75). Whether proprioceptive submodalities are similarly preprocessed in DCN complex nuclei remains unexplored (74, 76).

### Modulation of the Central Proprioceptive Stream

Ascending proprioceptive feedback is regulated by descending control at many levels, beginning in the peripheral muscle. Muscle spindles are subject to efferent motor control through dynamic and static gamma motor neurons that innervate the contractile polar ends of the intrafusal fibers and effectively set the gain for group Ia/II afferent discharge frequency (27, 77, 78). Proprioceptive feedback is also regulated by descending control through presynaptic inhibition of muscle spindle or GTO afferent terminals that contact the spinal second-order projection neurons (79, 80). In addition, descending inputs from cortical, reticulospinal, or vestibular supraspinal areas can directly control the excitability of proprioceptive projection neurons (56, 73, 81).

Proprioceptive streams within the DCN complex are modulated through descending input from sensory and motor cortices (44, 82, 83). With most neurons receiving modality-selective inputs (in contrast, see 83), the DCN complex is an important target for sensory gain modulation to influence which sensory features to strengthen and which to attenuate in higher processing (74, 75). With regard to the integration of proprioceptive and tactile inputs, descending modulation may favor proprioceptive over tactile feedback. Cortical input is largely excitatory to the rostral proprioceptive cuneate, but inhibitory to the middle cuneate, where most neurons respond to tactile stimuli (44, 66). Consistent with this observation, muscle afferent input is favored during active movement, whereas cutaneous input is repressed (74).

### Thalamus and Cortex

**Figure 3:**
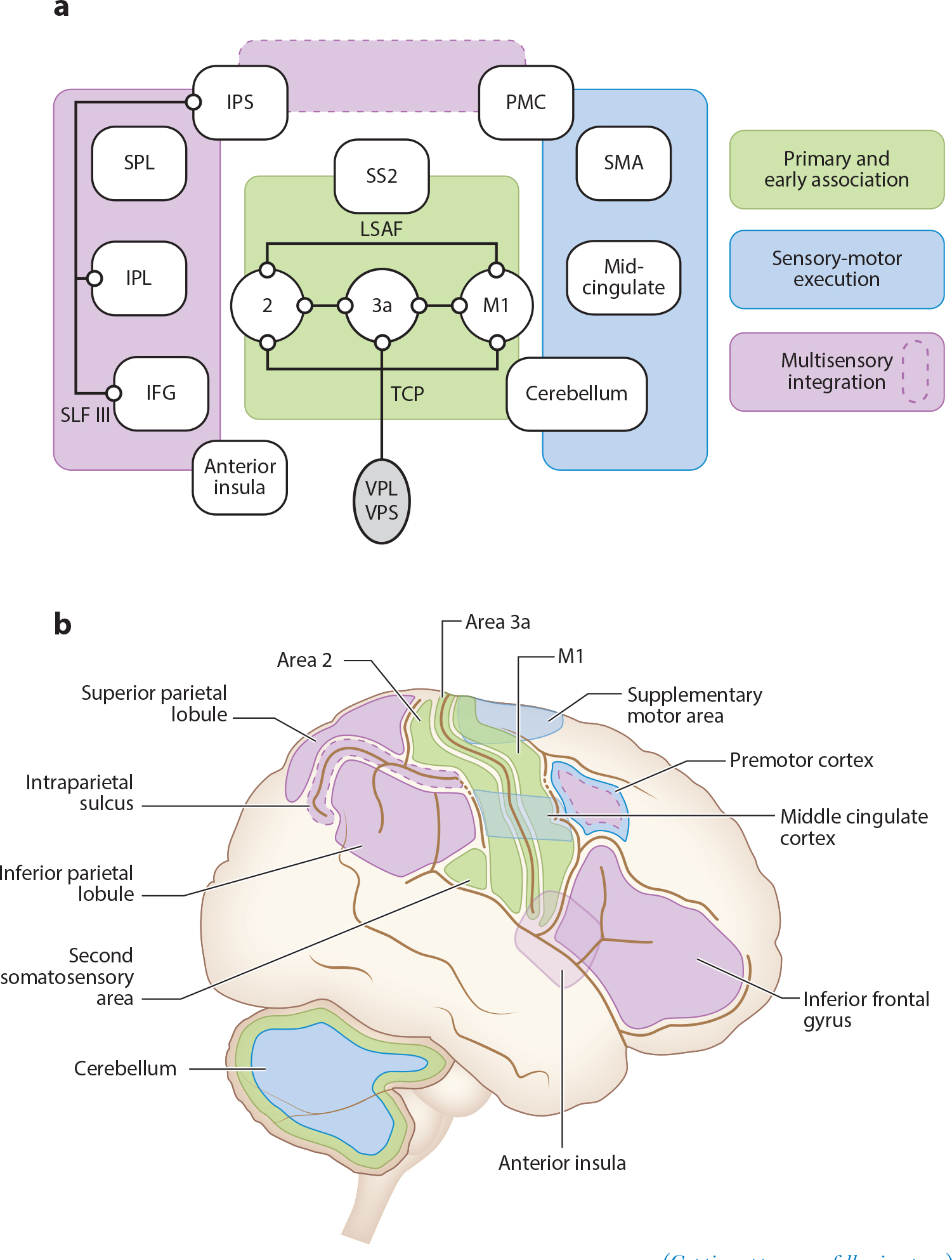
Cortical areas involved in kinesthetic sensation. (*a*) Schematic rendering of the cortical brain regions identified with imaging (functional magnetic resonance imaging and positron emission tomography) through the application of kinesthetic illusion-inducing vibration (70–115 Hz) to the wrist and forearm tendons of human participants. The brain regions are divided into three groupings. (*Orange*) The primary and early association areas including primary motor cortex (M1), area 2 (2), area 3a (3a), and second somatosensory area (SS2). Areas 3a, M1, and 2 are connected to the thalamus [ventroposterior lateral nucleus (VPL) and ventroposterior superior nucleus (VPS)] through the thalamocortical projection (TCP) fibers (165, 166). Areas 3a, M1, and 2 are also interconnected through local short association fibers (LSAF) (91). (*Blue*) A sensory-motor execution network including the premotor cortex (PMC), supplementary motor area (SMA), middle cingulate cortex (mid-cingulate), and cerebellum. (*Purple*) A likely higher-order multisensory integration system including the superior parietal lobule (SPL), inferior parietal lobule (IPL), intraparietal sulcus (IPS), inferior frontal gyrus (IFG), and anterior insula. The IPS, IPL, and IFG appear to be connected with the large superior longitudinal fasciculus III (SLF III) (102, 109). The cerebellum shares connectivity with the primary early association areas and the sensory-motor execution network. The IPS and PMC (*purple dashed outline*) are involved in a comparator network containing multimodality neurons that retune depending on visual and proprioceptive input with critical involvement in self-identification (113). (*b*) A schematic representation of the relative location of the brain areas and functional groupings of vibration-induced illusory kinesthesia in the human brain. Colors correspond to those in panel *a*.

Closer to cortex, the model system of choice tends to shift from rodents and cats to nonhuman primates and humans. All dorsal column nuclei project to both cerebellum and thalamus, but individual nuclei of the DCN complex exhibit different output preferences for each of the subcortical structures (70, 84, 85) (Figure 2b). Proprioceptive thalamic projections from the DCN complex are primarily directed to the ventroposterolateral (VPL) thalamic nucleus (69, 86). Information from lower trunk and hindlimb to thalamus (routed through nucleus Z, nucleus X, and rostral gracile) is mainly found in the VPL shell (87). From the VPL thalamic nucleus, proprioceptive afferents connect primarily to S1 somatosensory cortical areas 3a and 2 (88, 89), as well as to cortical area 4, also referred to as primary motor cortex (M1) (90). Primate studies have revealed that sensory areas 3a, 2, and M1 are highly interconnected (87) (Figure 3a). These interconnectivity results are corroborated in human diffusion tensor imaging brain area connectome analyses (91).

Following proprioceptive connectivity beyond the direct thalamo-cortical connections to sensory-motor primary and early association areas of the cortex becomes more challenging. Nevertheless, human brain imaging combined with vibration-induced illusory joint movements offers the potential of revealing important facets of proprioceptive organization across cortical association areas. Vibrating the tendons of skeletal muscles between 70 and 115 Hz generates illusory sensations of limb movement without the corresponding physical movement of the joint itself (92) (Figure 1c). The illusion of movement is strong enough to give the perception of limbs assuming impossible positions (93, 94), and the illusions can be used to produce sensation of complex three-dimensional arm movements by simultaneously addressing multiple joints (95). Using the kinesthetic illusion to amplify muscle sensory input provides an avenue to disambiguate the potential organizational differences between kinesthesia and confounds from the effects of action on perception [the efferent process (96)] through either executed or imagined movements. In accordance with electrophysiological results in primate studies, kinesthetic illusion-inducing vibration applied to the tendons of the wrist in humans shows brain activation, through positron emission tomography and functional magnetic resonance imaging, in areas M1, 2, cerebellum, and 3a (97–101).

The use of kinesthetic illusion-inducing vibration to reveal potential cortical network organization of proprioception provides evidence of at least two different ways that kinesthetic muscle sensation is handled in central networks (102). The first central network seems to align with a bilaterally distributed motor planning and execution focused system with apparent connections between M1, premotor cortex, supplementary motor area, middle cingulate cortex, and both sensory and motor areas of the ipsilateral cerebellum (98, 100–103) (Figure 3a). The organization of this network is further corroborated by electroencephalography and magnetoencephalography evidence from corticokinematic coupling experiments examining the relationship between sensory-motor brain activity and movement-related velocity signals from the peripheral proprioceptors (104, 105). Corticokinematic coupling shows brain activity in contralateral sensory-motor cortex (including the supplementary motor area), dorsolateral prefrontal cortex (overlapping with premotor cortex), posterior parietal cortex, and ipsilateral cerebellum (106–108). These regions are in alignment with the proposed vibration-induced illusory kinesthetic network suggested above. Similarly, the cingulate motor area, dorsal premotor cortex, supplementary motor area, and cerebellum are likely involved with possible neurophysiological mechanisms for comparing a neural proxy of motor intent commands (efference copy) with reafferent proprioceptive feedback (96).

The second central network appears to be a multisensory integration-focused system with relevant areas in lateral parietal lobe and frontal lobe, including inferior parietal lobule, secondary somatosensory area (SS2, OP1), anterior insula, and inferior frontal gyrus (102, 109) (Figure 3a). Vibration-induced kinesthetic illusory input specifically activates the right inferior frontoparietal brain areas (99, 110) that reside within the connective domain of the third branch of the superior longitudinal fasciculus (109). The connection of brain areas that are processing kinesthetic information from the muscles to a large fascicular network suggests the capacity for rapid communication between the relevant nodes within the inferior frontoparietal network (102). The speed of information transfer may be important for the online comparison between intent and sensations of resulting actions (e.g., comparison between expected and observed states, or action monitoring) (110). For example, the inferior parietal lobule and areas of the temporoparietal junction appear to monitor discrepancies between intent and multisensory feedback (including vision and proprioception) (110–112).

Multisensory integration is a complex and evolving concept in functional neural organization. Touch, hearing, vision, and proprioception converge in specialized networks centered in the parietal lobe that involve multiple cortical areas across different lobes of the brain (113, 114) (Figure 3b).The multisensory integration system appears to function as a Bayesian comparator to continuously monitor and correct for errors between the individual’s internal model of predicted reality and actual external reality as ascertained through the senses (115). Kinesthesia appears to play a central role in mediating the interaction between intent and outcome that is necessary for establishing a framework for self-reference (103) and seems to share a primary comparator subnetwork (premotor cortex–intraparietal sulcus) that is also important for visual-tactile integration and body ownership (113) (Figure 3a). The ability to artificially induce kinesthetic perception paired with methodologies for systematically modulating and controlling multiple feedback streams offers new ways to explore these systems within the context of advanced imaging and high-density electrophysiological recordings in humans. As methodologies develop, resolving the individual functional units within the multisensory integration system becomes more realistic.

## TOWARD AN UNDERSTANDING OF THE PROPRIOCEPTIVE SENSE THROUGH GENETIC ANALYSES

Advanced transcriptomics in mice presents opportunities to help resolve some of the questions raised in the prior sections. Genetic analyses of molecularly defined subsets of spinal projection neurons offer new insight into the dissemination of proprioceptive feedback to cerebellum and the DCN complex (24, 48, 116). Genetically empowered viral strategies paired with behavioral tests also enable the dissection of the DCN complex circuitry (66), and thalamic and cortex transcriptomic data are actively being mined and exploited for genetic interrogation (22, 117).

Until recently, the diverse sources of proprioceptive feedback made it challenging to probe individual receptors within the operational framework for the proprioceptive sense. This was mainly because it is difficult to assess the function of one class of afferents in isolation, especially under normal physiological conditions (i.e., in awake behaving animals or humans) (118). The reliance on electrophysiological analyses, while extremely informative, also presents limitations when seeking systematic insight into proprioceptive control systems. Now, however, with combined genetic tools, proprioceptive afferents can be marked with fluorescent proteins to facilitate their isolation from other sensory neurons in dorsal root ganglia (19, 20). Using these strategies in combination with advanced single-cell transcriptome analyses has permitted a detailed view of all the genes that are expressed in developing or mature proprioceptors. A subsequent comparison of single muscle afferent transcriptomes has enabled the identification of transcripts that distinguish between muscle spindle and GTO afferent subtypes. Such studies are offering new insights into the molecular underpinnings of the development and physiological properties of the individual proprioceptor subtypes (19, 20). Moreover, the differential expression of gene products selective for either muscle spindle or GTO afferent subtypes can serve as a foundation for a systematic genetic interrogation of their spinal and supraspinal targets or their roles in movement control (119). Similar strategies have already provided genetic access to individual types of cutaneous receptors (23, 36).

Proprioceptor transcriptome studies provided distinct molecular signatures for not only muscle spindle and GTO afferents but also spindle afferent subtypes. Instead of the expected group Ia and II muscle spindle afferent populations, transcriptional analyses in adult mice revealed multiple (perhaps as many as seven) distinct muscle spindle afferent subtypes (19, 20). Although the exact number of (molecularly) distinct muscle spindle afferent subtypes remains a topic of debate, these studies demonstrated that the diversity among muscle spindle afferent subtypes is larger than previously appreciated. Together these muscle proprioceptor transcriptional studies lead to two main findings. First, group Ib GTO afferents are represented by a single molecular class, suggesting that their feedback (e.g., information regarding muscle force) is relatively invariable, irrespective of peripheral muscle targets or intramuscular location (29). Second, muscle spindle afferents are represented by multiple different molecular subtypes. The diversity in muscle spindle afferent subtypes appears to further emphasize the importance of kinesthetic information in proprioception, as it suggests a need to be able to optimally tune this feedback.

A challenge for future studies is to understand the functional correlates of the different molecularly defined muscle spindle afferent subtypes. There may be a difference in physiological or circuit properties. The latter could include differences in contacts with intrafusal muscle fibers (chain, bag1, bag2), differences in the type of muscle targets (e.g., fast or slow fatigue, axial or limb), or differences in central targets (27). For example, it is not inconceivable that information from axial muscle may need to be distributed to different neural circuits than distal limb muscles, thus necessitating a molecular mechanism through which muscle spindle afferents can recognize the correct downstream targets. Indeed, when proprioceptors are molecularly profiled at earlier developmental time points (in embryos and neonates) they exhibit molecular identities that correlate with the type of muscle target they innervate (120–122). Conceivably, the newly recognized diversity in muscle spindle afferent subtypes may increase the complexity of an already complex sense. Nevertheless, genetic access to these individual receptors should help resolve some of the unexplained observations that result from the pooling of diverse sensory inputs.

## FUNCTIONAL DECONSTRUCTION OF PROPRIOCEPTIVE PERCEPTS

What is the relative importance of the various receptors for the proprioceptive sense? In other words, which information best serves the motor system to function optimally, with optimal defined as achieving the motor goal with the lowest energy expenditure yet highest sense of fulfilment (123)? The answers to these questions can be partially derived from animal studies (124, 125), patients afflicted by sensory neuropathies that impair proprioceptive neurons (40, 126, 127), functional imaging studies (102), and more recently, work with amputees with experimental prostheses that use bionic neural-machine interface strategies (9, 10, 17, 18). Two important observations are beginning to emerge from these studies: the relatively outsized role for dynamic kinesthetic feedback and the context-dependent relationship between exteroceptive (tactile) and interoceptive (proprioceptive) feedback in motor control. We explore these concepts in more detail below.

### The Kinesthetic Movement Sense

Kinesthetic information about limb movement and position is thought to derive primarily from group Ia and II muscle spindle afferents (2, 5, 25, 27). Group Ia muscle spindle afferents have low activation thresholds, are ranked among the somatosensory neurons with the fastest conduction velocities, and possess a high dynamic sensitivity. The latter underlies the 1:1 firing response of Ia muscle spindle afferents to vibratory inputs from 10 to ~100 Hz (92, 128). The dynamic sensitivity of Ia muscle spindle afferents enables them to signal muscle length and length change velocity (i.e., displacement). Group II muscle spindle afferents exhibit less dynamic sensitivity but higher discharge levels during the static phase of muscle stretch, suggesting that they are more reliable in encoding steady-state limb position than group Ia muscle spindle afferents (27, 129). Muscle spindle afferent activities are constantly measured against a central feedforward model of body/limb state such that changes in the predicted spindle afferent discharge frequencies serve as error detectors when a planned movement is perturbed and deviates from its intended trajectory. In addition, it is postulated that the difference in discharge between afferents of dedicated antagonistic muscle pairs is the basis for how the central nervous system computes relative limb position and movement (2).

Of the two proprioceptive senses (movement and position), the kinesthetic movement sense appears to provide the most significant input to proprioception. This is perhaps most powerfully demonstrated through vibration of muscle tendons, which generates illusory sensations of limb movement without a corresponding movement of the joint itself (see above) (92) (Figure 1c). The kinesthetic illusion is considered to be generated by the vibration-induced activation of group Ia primary muscle spindle afferents (92, 130). Afferents that innervate Pacinian corpuscle receptors in joints and deep tissues such as the interosseus membrane possess a similar dynamic sensitivity as observed for group Ia muscle spindle afferents (36, 37) (Figure 1a). However, because kinesthetic illusions can be generated when skin or joints are anesthetized or after lesions of the dorsal columns (through which the afferent information from Pacinian receptors is transmitted centrally), it is likely that Pacinian or other skin or joint afferents are not primary contributors to kinesthetic illusions (5). In addition, the degree of movement illusions are influenced by the prior activity (thixotropic conditioning) of the vibrated muscle and on the level of engagement of the fusimotor system (131–133). Thixotropy and fusimotor control are specific to muscle and muscle spindles, respectively, supporting the notion that the sensory origin of the kinesthetic sense is intrinsic to muscles.

Although it is undisputed that group Ia muscle spindle afferents can provide kinesthetic movement information, some observations raise the question of whether this is a property of all or just a subset of these afferents and/or whether there are other muscle afferents that can sense muscle vibration. For instance, the kinesthetic illusion is associated with a selective frequency bandwidth (~70–115 Hz), yet group Ia muscle spindle afferents are sensitive to vibration across the entire 1–100 Hz frequency range (92, 130). In addition, while some participants report the kinesthetic illusion following tendon vibration, they do not always exhibit the expected concomitant Ia reflex response that results in muscle contraction (134, 135). Similarly, reports of kinesthetic illusions are often difficult for participants to articulate and prone to priming, and they can switch direction (136–138). Taken together, these studies reveal the dominance of kinesthesia in the construction of proprioceptive percepts, yet they also raise questions about the mechanisms by which kinesthetic signals can outweigh other proprioceptive feedback streams during illusionary movements.

### Interactions Between Tactile and Proprioceptive Feedback

Proprioceptive feedback from cutaneous or joint receptors appears to serve a relevant supporting role in motor control (102) and perception of movement (139). Based on experiments coupling vibration-induced, muscle-sensory activation and skin stretch, it appears that the two information streams are continuously integrated and likely contribute to the identification of specific joint movements (138). For example, Collins et al. (138) stretched skin at the metacarpophalangeal joint while vibrating the corresponding digit tendons on the dorsum of the hand, which separated stretch from the vibrational input and likely aided in potentiating the synergistic kinesthetic percepts. Similarly, if care is taken to couple vibration at the residual tibialis anterior with skin stretch on the dorsum of the knee, individuals with below-knee amputations can have enhanced perception of illusory joint movements (140). However, simply vibrating muscle tendons through the intact skin of the corresponding joint often leads to ambiguity and confusion when reporting movement illusions (137, 141).

Tactile feedback appears to supersede proprioceptive information in the conscious perception of sensation. For instance, kinesthetic illusions are diminished when there is direct tactile (or visual) feedback of the arm with the vibrated muscle. This suggests that when multiple feedback systems are operating simultaneously the brain will integrate, but sometimes also prioritize, potentially conflicting information from other feedback systems (142, 143). However, neural-machine interfaces in individuals with amputation may help to provide insight into the synergistic (or potentially antagonistic) relationship between sensory receptors in the skin and sensory receptors in the muscle (see also below).

## INSIGHTS INTO PROPRIOCEPTIVE PROCESSING THROUGH THE USE OF APPLIED PROPRIOCEPTIVE FEEDBACK IN ADVANCED PROSTHETICS

Targeted reinnervation is a technique that provides intuitive motor control and sensation of touch and joint movement for advanced prosthetic limbs to individuals with amputation (10, 144, 145). The sensory-motor feedback and control interface is created by surgically redirecting the amputated limb nerves to new proximal muscle and skin sites. The redirected nerves reinnervate purposely denervated target muscles and target skin to provide biological amplifiers for the neural motor control signals and sensory interfaces for neurorobotic touch and kinesthesia. Importantly, with targeted reinnervation, the natural correspondence between the skin of the joints and their underlying muscle and tendon is altered through the surgical sensory-motor reassignment of the amputated muscle and skin nerves (17). With this approach, it is possible to address kinesthesia and touch individually.

### Kinesthetic Perception in a Neural-Machine Interface

Vibrating the neurally reassigned muscles to induce kinesthetic illusions reveals highly complex synergistic hand and finger movements that reflect functionally relevant grip conformations such as cylinder grip, tripod grip (three-jaw chuck), fine pinch, and flat hand pinch (17). Uniquely, all study participants consciously reported similar percepts. Interestingly, the illusory percepts reflect contraction, not elongation, of the muscles activated by vibration. Muscles reinnervated by the median nerve provide clear percepts of finger/joint flexion when vibrated, whereas the muscles reinnervated by the radial nerve provide clear percepts of finger/joint extension (17). These percepts are the opposite of what would be predicted by sensory feedback of movement occurring through activation of receptors sensitive to muscle stretch (Figure1c). This contrary finding is also corroborated in neural-machine interfaces using electrical stimulation as the feedback modality, where activation of the median nerves produces percepts of digit flexion (12, 146).

Although input from cutaneous joint receptors likely plays a central role in perception of movement, kinesthesia seems to play a greater role in informing the function of motor control systems. For example, prosthetic touch alone, provided through various neural-machine interfaces, improves function over insensate prosthetic limbs when identifying and grasping objects (8, 147–152). However, providing kinesthetic perception allowed prosthetic users to achieve even greater able-bodied level performance in a grasp prepositioning task (17). Similarly, participants showed substantive improvements in achieving evenness of grip closure proportionality (four equal divisions), their ability to track a moving grip aperture target, and their ability to adapt to intrinsic error, even in the presence of vision (17). Interestingly, participants provided with kinesthetic sensation alone were largely unaware of the improvement to their prosthetic usage. They did not perceive the kinesthetic feedback as contributing to the effective use of their prosthetic device although their testing results clearly demonstrated high levels of function with no training. When the kinesthetic sensation was on, their performance was improved, in some instances to the level of able-bodied function on the same task. When kinesthetic sensation was turned off, the effect was abolished and they performed at levels reflecting basic prosthetic users. Together, these observations support the primacy of muscle-mediated kinesthetic feedback in movement control but also suggest that it operates largely outside of conscious perception. Kinesthesia likely provides online feedback to the internal model for more effective motor integration. This hypothesis was corroborated in a study where amplifying kinesthetic feedback of the contracting muscles through vibration-induced perceptual illusions improved reaching and pointing performance inindividuals with central sensory area strokes (153).

### The Integration of Kinesthetic and Tactile Modalities

Neural-machine interfaces offer additional insight into the relationship between kinesthesia and cutaneous tactile sensation. Decoupling kinesthetic sensation from joint-mediated cutaneous sensation and then fusing kinesthetic feedback to the contextually appropriate touch events (contact transients and proportional pressure) and motor intent returns natural behaviors to individuals with amputation (10). The interaction between grip kinesthesia and proportional fingertip pressure feedback allowed for a naturally balanced decision strategy (reflecting able-bodied behavior) on a sorting task based on object durometer. With integrated touch and kinesthesia, participants struck a natural balance between completing the sorting task quickly and taking the time to make as few mistakes as possible. Furthermore, providing contextually relevant touch and kinesthetic feedback also released the participants from using vision to control their devices. In a reflection of natural able-bodied behavior, they could look ahead toward the site of object placement in anticipation of their next planned action. Again, these improvements in performance occurred without learning. When the integrated system was on, the participant’s behaviors stratified with able-bodied functionality. When the integrated systems were off, the participants stratified with typical prosthesis users.

Similar returns to natural reflexive behaviors without training are also seen in a proprioceptive neural-machine interface based on coupling the agonist–antagonist relationship through a capstan pulley system surgically constructed from muscles and tendons during amputation (18). The agonist–antagonist myoneural interface system restores the lost reciprocal relationship between contracting and elongating muscles. As such, participants were able to reflexively modulate ankle joint angles in the proper context while climbing and descending stairs and also while intentionally everting their prosthetic foot to account for stepping on the edge of a small block. The two neural-machine interface systems may reveal potential mechanistic differences between the traditional group Ia muscle spindle–mediated system of torque feedback in the antagonist myoneural interface and the vibration-induced readout of active contraction of the agonist muscle in targeted reinnervation. Further comparison between successfully implemented proprioceptive interfaces provides the opportunity to resolve the facets of proprioceptive sensation that are most relevant to function. Implementing these systems in human participants with amputation allows for insight from the users to help answer questions, such as, what is the relationship between perception and nonconscious utilization? Does the individual need to be in the loop perceptually, or are their function and utilization more effective when they occur outside of perception and do not require their attention? Furthermore, work in humans with neural-machine interfaces suggests that there are different modes of proprioceptive sensation that provide different functional improvements. These bionic systems could be used to guide new investigations into a poorly understood sensory system.

## MULTISENSORY INTEGRATION AND THE SENSE OF AGENCY

In humans, second somatosensory area (SS2/OP1) is considered to be a center for motor and multisensory integration (Figure 3a), including not only proprioception and touch but also vision and pain (154). In corroboration with human studies (99), electrophysiological recordings in the rodent transitional zone (the rodent homolog to second somatosensory area) show specific cortical multiunit kinesthetic responses from muscle triggered by vibratory frequencies that are in alignment with the vibration-induced kinesthetic illusion in humans (155). Considering the second somatosensory area as a confluence point for complex multisensory and motor information, it has been proposed that this brain area may also be involved with a somatocentric (possibly perceptual) mapping of the body in contextual relationship to its external environment (156, 157).

Multisensory integration and intent/action/outcome monitoring are key aspects of the ability to discern whether intended actions result in their predicted outcomes. The sense of agency arises from the experience of being in control of one’s actions and is often attributed to a comparator mechanism that uses efference copy to reference an internal predictive model of movement in comparison to sensory feedback arising from the movement itself (103). There appear to be two primary subcomponents of agency. The first is a system for detecting unexpected external events that are out of alignment with internal models (nonagency). The inferior frontoparietal network, described previously, is a likely neural corollary for this proposed nonagency system (103, 158). The second system relates to a retrospective inference about one’s own control over their actions (self-agency) (103,158).The concept of self-agency, or the experience of being the author of one’s actions (159), is less well understood. However, the anterior insula (Figure 3a) is activated with kinesthetic illusion-inducing vibratory input (99, 102, 109) and is frequently implicated in studies that specifically address the experimental induction of self-attribution (158). Overall, the insula appears to be a brain region that serves as a functional integration center for multiple cognitive and perceptual processes, whereas the anterior insula is considered to be involved with the awareness of causing an action, general self-awareness, and time perception (160–162).

The sense of agency is intertwined with the sense of body ownership (the feeling of our body and body parts as belonging to oneself) (159). Several of the brain areas that appear to use kinesthetic sensory information to build the cohesive experience of controlling one’s actions (inferior parietal sulcus, superior parietal lobule, premotor cortex, temporoparietal junction, and insula) overlap with dorsal frontoparietal multisensory integration areas that help mediate the experience of body ownership (113) (Figure 3a,b). Future work will help to determine how the brain mechanisms of proprioception-mediated agency and visual-tactile-mediated body ownership combine and interact to establish a multisensory integration-derived perception of the embodied self (the experience of owning and controlling one’s body) (113).

Neural-machine interfaces utilizing vibration-induced kinesthetic feedback can be used to modulate the sense of agency (159). When prosthesis user intent is matched to visual and kinesthetic feedback of the intended movement, a sense of agency over actions is reported (17).There is a certain malleability of attribution of agency where the visual percept can differ slightly (faster or slower) from the perceived sensation of movement. However, visualized hands that move contrary to intent and kinesthetic feedback (either moving in the opposite direction or moving with a 0.5-s delay) do not generate a sense of agency over the movement. The effective alignment between the internal predictive model and the observed outcome of the action (kinesthetic and visual) is a key component of the sense of agency. Interestingly, kinesthesia does not appear to provide a sense of ownership on its own (17). The concepts of agency and ownership appear to be two different multisensory integration mechanisms that combine together to form the overall sense of the embodied self.

## SUMMARY

In this review, we have provided a view on the state of the proprioceptive field with a look toward the future, highlighting the potential of bionic prosthetic devices and genetics to explore some of the many outstanding questions. With respect to the latter, we expect that the largest gains will be made at the circuitry level, with new genetic tools enabling a systematic dissection of the central pathways of the different types of proprioceptive afferents and spinal projection neurons, all the way through the DCN complex, cerebellum, and thalamus to the cortex. Better genetic tools for proprioceptive and tactile circuit elements should also facilitate high-resolution mapping of the intersection of these two modalities and testing their hierarchical relationship in diverse behavioral contexts. Increasingly clever methods for quantitative natural behavioral assays, combined with large-scale recordings, should further aid in these studies (66, 163, 164) and should be immensely valuable in generating and testing new hypotheses about the basic principles of proprioceptive encoding. We anticipate that many of these observations may also translate to nonhuman primates and humans, despite some of the differences in circuitry. The experiences with bionic prostheses in human participants, combined with imaging and electrophysiological approaches, likely will continue to offer unique insights into the cortical areas involved in conscious and nonconscious proprioceptive processing. In particular, the realization that kinesthetic information plays a central role in the establishment of the sense of self-versus-other and the feeling of the whole self that is owned and controlled by the individual will help pave the way to explore these critical concepts through neural-machine interfaces in humans.
